# Ferroptosis in ischemic stroke: mechanisms, pathological implications, and therapeutic strategies

**DOI:** 10.3389/fnins.2025.1623485

**Published:** 2025-11-11

**Authors:** Zhangling Long, Ying Zhu, Heng Zhao, Shuang Liao, Cuiying Liu

**Affiliations:** 1 Department of Neurology, Tongren People’s Hospital, Tongren, Guizhou, China; 2 School of Nursing, Capital Medical University, Beijing, China; 3 Beijing Institute of Brain Disorders, Laboratory of Brain Disorders, Ministry of Science and Technology, Collaborative Innovation Center for Brain Disorders, Capital Medical University, Beijing, China

**Keywords:** ferroptosis, ischemic stroke, lipid peroxidation, iron dysregulation, neuroprotection

## Abstract

Ferroptosis, an iron-dependent form of cell death driven by lipid peroxidation, has emerged as a pivotal mechanism in the complex pathophysiology of ischemic stroke, a leading cause of global death and disability. This review synthesizes current understanding of the core ferroptosis pathways, including iron dysregulation, glutathione depletion, and GPX4 inactivation and distinguishes it from other cell death modalities. We critically explore its role as a pathogenic amplifier in stroke, synergizing with neuroinflammation and mitochondrial dysfunction to expand neuronal injury. The review systematically assesses therapeutic strategies, from iron chelators and lipid peroxidation inhibitors (e.g., ferrostatin-1) to emerging gene therapies and nanomedicine-based approaches, based on robust preclinical evidence. However, translating these findings faces challenges, including a narrow therapeutic window, patient heterogeneity, and the need to balance efficacy with safety concerning systemic iron and lipid metabolism. To overcome these translational challenges, future research must prioritize the discovery of clinical biomarkers (e.g., FABP5) and the development of targeted delivery systems to advance ferroptosis-directed therapies for stroke.

## 1 Introduction

Stroke remains the second-leading cause of death and disability worldwide, with ischemic stroke accounting for approximately 80% of cases (Huang, 2023). Despite significant advances in acute interventions including thrombolysis and thrombectomy, therapeutic efficacy is constrained by both narrow time windows and heterogeneous patient responses (Xiong et al., 2022). The pathophysiological cascade following ischemic injury progressively involves energy failure, excitotoxicity, oxidative stress, and inflammation, ultimately culminating in irreversible neuronal death (Zhao, 2022). Among the diverse cell death modalities implicated in stroke pathogenesis, ferroptosis-an iron-dependent programmed cell death pathway characterized by lipid peroxidation-has emerged as a critical driver of ischemic neurodegeneration (Guo et al., 2023).

First described in 2012, ferroptosis is characterized by mitochondrial cristae shrinkage, glutathione peroxidase 4 (GPX4) inactivation, and lethal lipid peroxide accumulation through iron-dependent Fenton reactions (Li, 2020). In ischemic stroke, dysregulated iron metabolism and reactive oxygen species (ROS) overproduction synergistically amplify ferroptosis pathways, exacerbating neuronal injury (Guo et al., 2023; Manzanero et al., 2013). Elevated labile iron pools-primarily derived from hemoglobin degradation and disrupted iron transport-catalyze Fenton reactions, generating hydroxyl radicals that propagate lipid peroxidation and membrane destabilization (Liu et al., 2022; Liang et al., 2022). Concurrently, impaired antioxidant systems, particularly the glutathione/GPX4 axis, fail to neutralize lipid hydroperoxides, thereby accelerating ferroptosis cell death (Ursini and Maiorino, 2020).

Emerging evidence identifies ferroptosis as a maladaptive response in stroke, with its unchecked activation exacerbating infarct volume expansion and worsening functional outcomes (Chen, 2023a). Preclinical studies have demonstrated that pharmacological inhibitors of ferroptosis, such as ferrostatin-1 and liproxstatin-1, attenuate neuronal loss and enhance recovery (Du and Guo, 2022). Clinically, elevated levels of lipid peroxidation biomarkers including malondialdehyde (MDA) correlate strongly with stroke severity and poor prognosis (Lorente, 2015). However, the field is marked by both compelling evidence and perplexing contradictions. For instance, the same molecule may exhibit opposing roles in ferroptosis regulation across different cellular or disease contexts, and the primacy of specific pathways in human stroke remains debated. This review, therefore, moves beyond a mere summation of findings. It aims to critically evaluate the mechanistic contributions of ferroptosis to ischemic stroke pathophysiology, dissect the apparent discrepancies in the literature and assess emerging therapeutic strategies through a translational lens that highlights both promises and unresolved challenges.

## 2 Mechanisms of ferroptosis

Ferroptosis is a distinct form of regulated cell death characterized by the accumulation of lipid peroxides to lethal levels (Dixon and Olzmann, 2024). Unlike traditional cell death modalities such as apoptosis and necrosis, ferroptosis exhibits unique morphological and biochemical features (Bertheloot et al., 2021). Apoptosis, a well-defined process of programmed cell death, is characterized by caspase activation, chromatin condensation, and DNA fragmentation (Bertheloot et al., 2021). In contrast, ferroptosis is marked by mitochondrial shrinkage, increased membrane density, and the absence of apoptotic features such as chromatin condensation and apoptotic bodies (Li, 2020; Feng, 2024). On the other hand, necrosis, often resulting from acute cellular injury, leads to membrane rupture and subsequent inflammatory responses (Ketelut-Carneiro and Fitzgerald, 2022). In contrast, ferroptosis proceeds in a more regulated manner without the uncontrolled release of cellular contents typically seen in necrosis.

**Figure 1:**
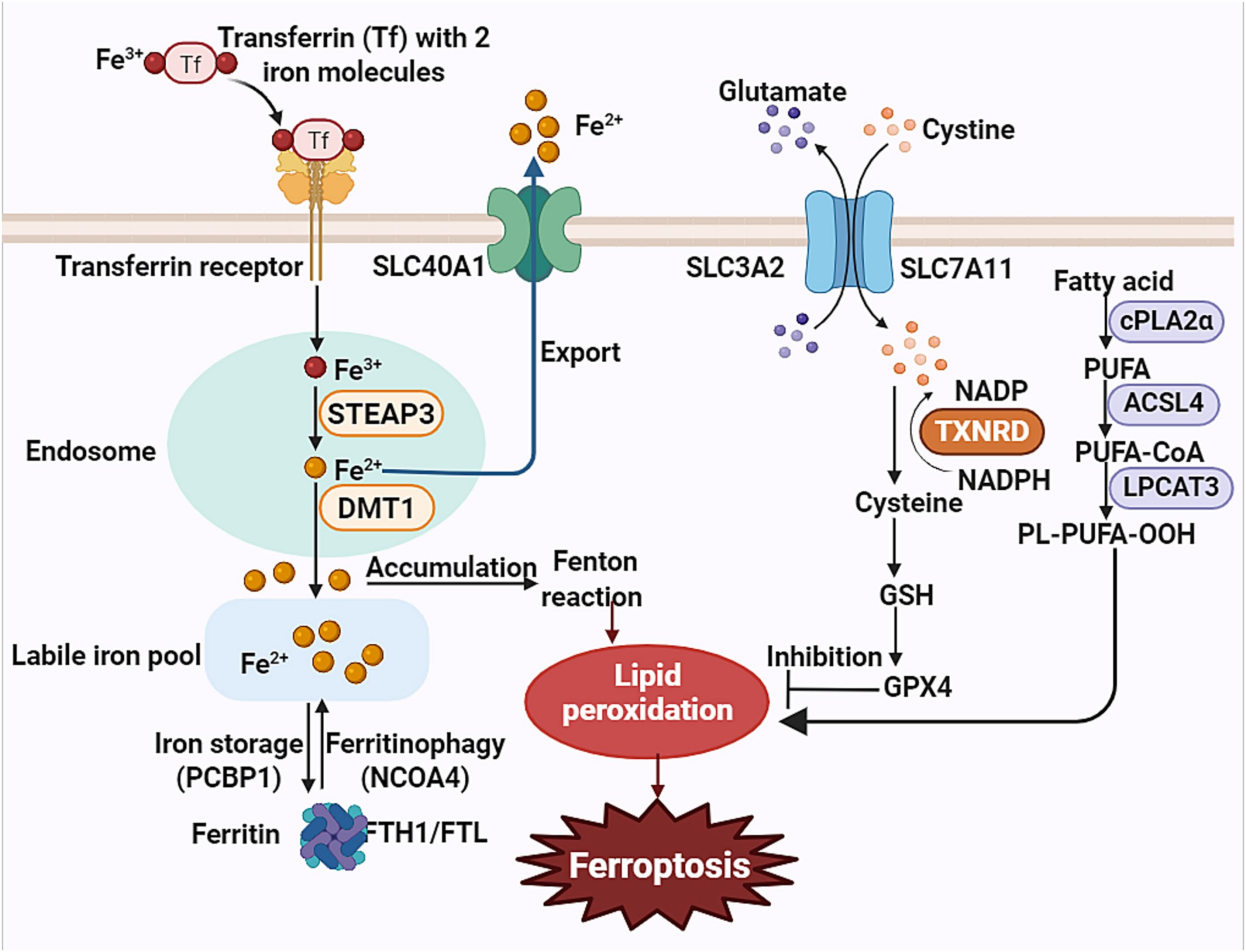
Mechanism of ferroptosis. This schematic delineates the core pathways driving ferroptosis. Transferrin (Tf) delivers ferric iron (Fe^3+^) into cells. Six-transmembrane epithelial antigen of the prostate 3 (STEAP3) reduces Fe^3+^ to ferrous iron (Fe^2+^), which is transported into the cytoplasm via divalent metal transporter 1 (DMT1). Excess Fe^2+^ catalyzes lipid peroxidation through Fenton reactions. Polyunsaturated fatty acids (PUFAs) are esterified into membrane phospholipids by acyl-CoA synthetase long-chain family member 4 (ACSL4) and lysophosphatidylcholine acyltransferase 3 (LPCAT3), forming peroxidation-susceptible phospholipids. Reactive oxygen species (ROS) directly oxidize PUFA-containing phospholipids, propagating peroxidation. The cystine/glutamate antiporter (SLC7A11) imports cystine for glutathione (GSH) synthesis. GSH is required by glutathione peroxidase 4 (GPX4) to neutralize lipid peroxides. Dysfunctional SLC7A11 reduces cystine uptake, depletes GSH, and inactivates GPX4, disabling cellular antioxidant defenses.

The hallmark of ferroptosis lies in the peroxidation of polyunsaturated fatty acids within cellular membranes, driven by an imbalance between ROS and the cells’ antioxidant defense systems (Liang et al., 2022; Figure 1). Under conditions of oxidative stress, ROS initiate the chain reaction of lipid peroxidation, culminating in the generation of toxic lipid hydroperoxides that compromise membrane integrity and cellular viability (Su, 2019). Key enzymes such as lipoxygenases (LOXs) catalyze polyunsaturated fatty acids (PUFAs) oxygenation into peroxides, while acyl-CoA synthetase long-chain family member 4 (ACSL4) primes ferroptosis by activating PUFAs (Mashima and Okuyama, 2015; Ding, 2023). Importantly, the activity of LOXs and the extent of lipid peroxidation are tightly linked to intracellular iron levels, as iron acts as a critical cofactor in the Fenton reaction, generating highly reactive hydroxyl radicals that amplify oxidative damage (Blokhina et al., 2003). When the accumulation of lipid peroxides surpasses the cellular threshold for detoxification, ferroptosis is irreversibly triggered.

Iron metabolism is central to the regulation of ferroptosis, as iron plays dual roles in cellular physiology: it is essential for oxygen transport, DNA synthesis, and enzymatic reactions, yet its dysregulation drives pathological lipid peroxidation (Rochette, 2022). Intracellular iron homeostasis is tightly regulated through a multi-step process. Iron uptake primarily occurs via transferrin receptor 1 (TfR1)-mediated endocytosis, where transferrin-bound iron (Fe^3+^) is reduced to Fe^2+^ by six-transmembrane epithelial antigen of the prostate 3 (STEAP3) and released into the cytosol (Kawabata, 2019). Excess iron is either stored in ferritin-a protein complex composed of ferritin heavy chain (FTH1) and light chain (FTL)-or exported via ferroportin (SLC40A1), the sole mammalian iron exporter (Galy et al., 2024; Donovan, 2005). This export process is modulated by hepcidin, a liver-derived hormone that regulates ferroportin activity in response to systemic iron levels and inflammation (Nemeth and Ganz, 2021; Wang and Babitt, 2019). Pathological iron overload arises when FTH1/FTL function is disrupted, leading to reduced iron sequestration and expansion of the labile iron pool (LIP) (Chen, 2020). The redox-active iron in the LIP fuels the Fenton reaction, generating hydroxyl radicals that amplify lipid peroxidation (Yu, 2021). Elevated levels of free iron thus sensitize cells to ferroptosis, particularly in conditions of oxidative stress or impaired antioxidant defenses. The glutathione (GSH)/GPX4 axis serves as the primary defense against lipid peroxidation (Ursini and Maiorino, 2020). GPX4 detoxifies lipid hydroperoxides using GSH as a cofactor, while GSH synthesis depends on cysteine imported via the solute carrier family 7 member 11 (SLC7A11) subunit of the system x_c_^−^ transporter (Shi, 2021; Li, 2022). Under metabolic stress, the tumor suppressor TP53 exacerbates ferroptosis by transcriptionally repressing both SLC7A11 and GPX4, thereby crippling cysteine uptake and GSH-dependent antioxidant activity (Gong, 2022). When this system fails, lipid peroxides accumulate irreversibly, culminating in ferroptosis.

In addition to lipid peroxidation and iron metabolism, several other pathways contribute to ferroptosis regulation. The ferroptosis suppressor protein 1 (FSP1)/Coenzyme Q10 (CoQ10) axis operates independently of GPX4, mitigating ferroptosis by reducing lipid radicals and regenerating antioxidants (Li W., 2023). Similarly, the GCH1/BH4 pathway modulates ferroptosis through the production of tetrahydrobiopterin (BH4), a cofactor that selectively protects membrane phospholipids from peroxidation (Hu, 2022). These interconnected pathways underscore the complexity of ferroptosis regulation and highlight its integration into broader cellular metabolic networks.

## 3 Ferroptosis as a pathogenic amplifier in ischemic stroke

### 3.1 Mechanisms of ferroptosis in ischemic stroke

**Figure 2:**
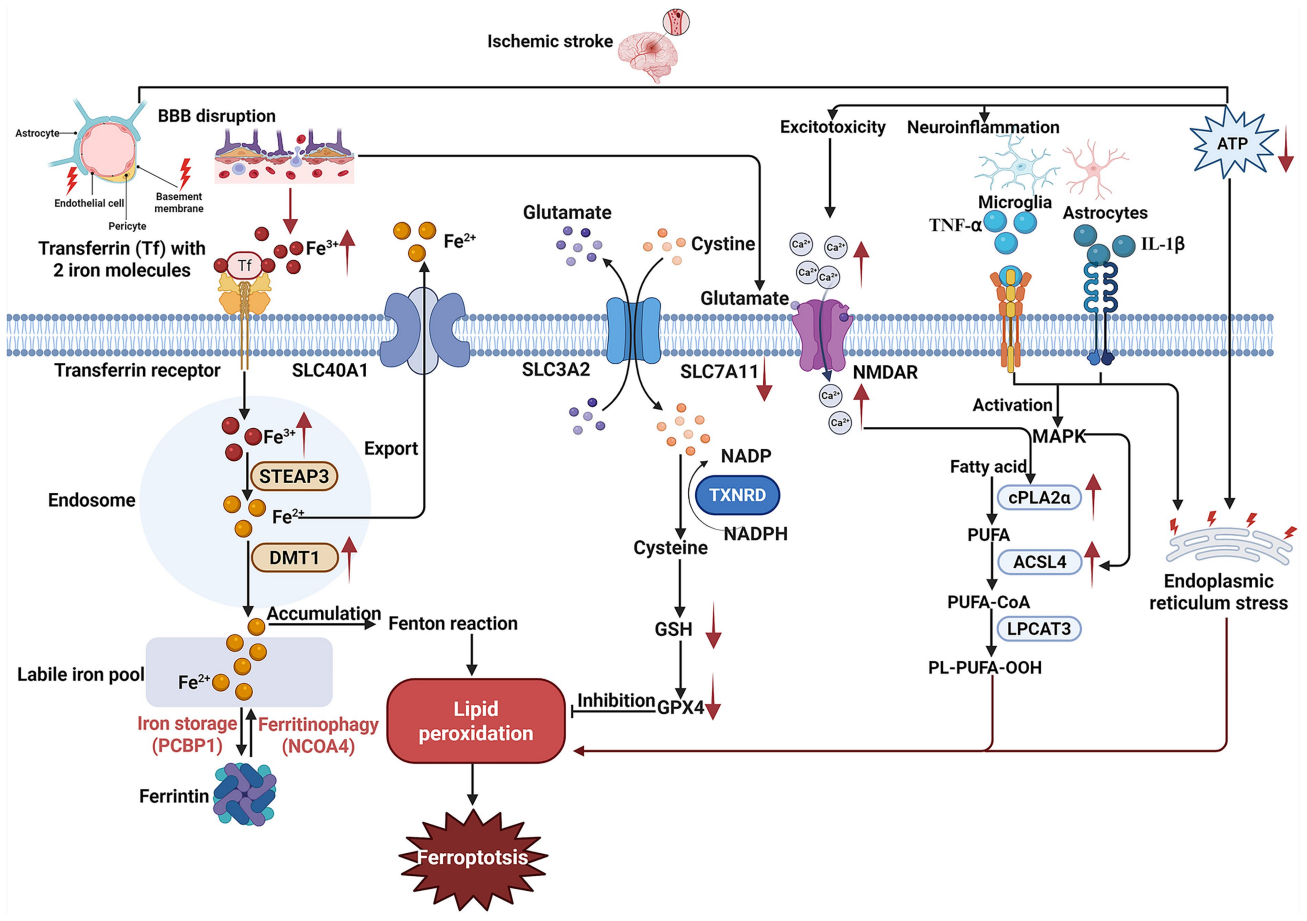
Mechanisms of ferroptosis in ischemic stroke. This schematic illustrates the pathophysiological cascade of ferroptosis following ischemic stroke. Transferrin (Tf) transports ferric iron (Fe^3+^) into brain endothelial cells. Blood–brain barrier (BBB) damage exacerbates iron accumulation, where excessive intracellular ferrous iron (Fe^2+^) drives Fenton reactions, generating hydroxyl radicals that initiate lipid peroxidation. Excitotoxicity-induced calcium influx activates cytosolic phospholipase A2α (cPLA2α), which hydrolyzes membrane phospholipids to release polyunsaturated fatty acids (PUFAs). These PUFAs are esterified into peroxidation-susceptible phospholipids by acyl-CoA synthetase long-chain family member 4 (ACSL4) and lysophosphatidylcholine acyltransferase 3 (LPCAT3), amplifying oxidative damage. Impaired cystine/glutamate antiporter (SLC7A11) function reduces cystine uptake, depleting glutathione (GSH) synthesis and inactivating glutathione peroxidase 4 (GPX4), which fails to neutralize lipid peroxides. Concurrent NADPH depletion compromises the thioredoxin reductase (TXNRD)-mediated antioxidant defense. ATP depletion activates microglia and astrocytes, triggering the release of pro-inflammatory cytokines. These inflammatory signals synergize with endoplasmic reticulum (ER) stress to exacerbate lipid peroxidation, ultimately culminating in ferroptosis.

Ferroptosis intensifies ischemic injury through mechanisms involving iron overload, lipid peroxidation, and mitochondrial dysfunction (Tuo and Lei, 2024) (Figure 2). During cerebral ischemia, disrupted iron homeostasis leads to the accumulation of labile iron, which catalyzes Fenton reactions and generates hydroxyl radicals (Guo et al., 2023). These radicals initiate lipid peroxidation of PUFAs in neuronal membranes, resulting in the production of cytotoxic aldehydes, such as 4-hydroxynonenal (4-HNE) and MDA, which disrupt membrane integrity and organelle function (Delgado-Martín and Martínez-Ruiz, 2024; Ayala et al., 2014). The inactivation of GPX4, a hallmark of ferroptosis, further depletes antioxidant defenses, permitting the unchecked accumulation of lipid peroxides (Wei, 2024). Mitochondrial dysfunction exacerbates ferroptosis injury (Pedrera, 2025; Tian, 2024). Ischemic stress induces mitochondrial membrane depolarization, ATP depletion, and ROS overproduction, creating a vicious cycle that amplifies lipid peroxidation (Huang, 2023). Additionally, endoplasmic reticulum (ER) stress triggered by ischemia activates the unfolded protein response (UPR), which intersects with ferroptosis via the IRE1α-XBP1 axis (Yang, 2024; Li Y., 2024).

Ferroptosis synergizes with neuroinflammatory pathways to exacerbate ischemic damage (Chen, 2023b). Dying neurons release damage-associated molecular patterns (DAMPs), which activate microglia and astrocytes (Lin M. M., 2022). In glutamatergic neurons, ferroptosis-driven GPX4 dysfunction and mitochondrial collapse amplify excitotoxicity, while astrocytes lose their ability to regulate glutamate or maintain the blood–brain barrier (BBB), exacerbating edema (Jacquemyn et al., 2024; Mahmoud, 2019). Ferroptosis cells secrete pro-inflammatory cytokines, such as tumor necrosis factor-alpha (TNF-*α*) and interleukin-1 beta (IL-1β), along with ROS, leading to disruption of the BBB, recruitment of peripheral immune cells, and propagation of secondary injury (Chen, 2023b). Infiltrating macrophages and activated microglia shift to pro-inflammatory states, further amplifying oxidative stress and releasing DAMPs that trigger NOD-, LRR- and pyrin domain-containing protein 3 (NLRP3) inflammasome activation (Zhan, 2022). Additionally, ferroptosis disrupts mitochondrial energy metabolism in microglia, impairing their ability to clear apoptotic debris via efferocytosis (Liu Z., 2025). Accumulated cellular debris perpetuates DAMP release, activating the NLRP3 inflammasome and caspase-1-dependent pyroptosis in neighboring cells, thereby expanding the ischemic penumbra (Zhan, 2022). Oligodendrocytes contribute to white matter damage through iron-dependent oxidation of myelin lipids, while endothelial cell ferroptosis disrupts vascular integrity, facilitating leukocyte infiltration (Shen, 2022; Lin X., 2022). Collectively, these cross-amplifying mechanisms drive neuroinflammation, neuronal death, and tissue destruction, ultimately worsening neurological outcomes.

### 3.2 Evidence of the effect of compounds as targeted therapies that reduce ferroptosis in ischemic stroke

The role of ferroptosis in ischemic stroke has been well-documented in experimental studies. Pharmacological ferroptosis inhibitors have been shown to reduce infarct size and improve neurological outcomes in stroke models by targeting lipid peroxidation and disrupting the vicious cycle of oxidative stress (Du and Guo, 2022). However, a deeper dive into the literature reveals a complex and sometimes contradictory landscape of molecular regulators, which must be critically appraised to guide future research and therapy development.

Accumulating evidence supports the therapeutic targeting of ACSL4 protein stability to modulate ferroptosis sensitivity. ACSL4 exacerbates ischemic stroke via two parallel mechanisms: triggering neuronal ferroptosis via lipid peroxidation and fueling neuroinflammation, the latter being evidenced by suppressed cytokine production upon its downregulation in microglia (Cui, 2021). Melatonin mitigates ferroptosis and neuronal damage in ischemic stroke models, an effect achieved through the MDM2-mediated ubiquitination and degradation of ACSL4, leading to a reduction in ferroptosis markers like intracellular Fe^2+^ (Yu, 2024; Ji et al., 2024). This finding highlights ACSL4 as a promising target. Tongqiao Huoxue Decoction (TQHX) confers neuroprotection against cerebral ischemia–reperfusion injury by promoting the ubiquitin-dependent degradation of ACSL4 (Ou, 2024). Collectively, these findings indicate that regulating ACSL4 stability is a viable anti-ferroptotic strategy.

A significant challenge in the field is the context-dependent functionality of several key players. In mouse tumor xenograft models, Glutaredoxin 5 (GLRX5) was shown to mediate ferroptosis (Lee, 2020). Interestingly, the regulation of this pathway appears to be context-dependent. A study by Zhou et al. demonstrated that *Salvia miltiorrhiza* Bge processed with porcine cardiac blood alleviated cerebral ischemia–reperfusion injury by inhibiting GLRX5-mediated ferroptosis (Zhou S., 2024). Conversely, Liu et al. found that exosomes from hypoxic pretreated ADSCs attenuated ultraviolet light-induced skin injury by delivering GLRX5 to inhibit ferroptosis (Liu, 2024). This apparent contradiction underscores that the role of molecules like GLRX5 may not be absolute but is probably shaped by cell type, subcellular localization, and the nature of the insult. It cautions against the oversimplified classification of targets as purely “pro-” or “anti-ferroptotic” and emphasizes the need for cell-type-specific mechanistic studies.

Ferroptosis is not an isolated event but a coordinated pathology across the neurovascular unit. Evidence now shows that ferroptosis occurs simultaneously in multiple cell types. In a rat focal ischemia model, Ginsenoside Rd. (G-Rd) preserved blood–brain barrier integrity by activating the NRG1/ErbB4 signaling pathway to enhance tight junction protein (ZO-1, occludin, claudin-5) expression and reduce endothelial cell loss, and by inhibiting endothelial cell ferroptosis (Hu, 2024). Cottonseed oil (CSO) treatment was shown to protect BBB integrity, reduce iron influx, and inhibit ferroptosis in middle cerebral artery occlusion (MCAO) models (Sun, 2023). Piceatannol protects against cerebral ischemia or reperfusion injury by inhibiting neuronal ferroptosis through the USP14/GPX4 axis (Zhao, 2025), and ACSL4 in microglia fuels neuroinflammation (Cui, 2021). The integration of these findings reveals a devastating positive feedback loop: neuronal ferroptosis releases DAMPs that activate microglia, whose inflammatory response further sensitizes neurons and endothelial cells to ferroptosis. This paradigm shift argues that the most effective therapies will be those that can disrupt this intercellular vicious cycle, either through multi-targeted agents like L-F001 or combination regimens (Peng, 2022).

The identification of fatty acid-binding protein 5 (FABP5) as a specific biomarker of ferroptosis in damaged human neurons marks a significant advance (Peng, 2024). FABP5 is uniquely upregulated and stabilizes at the cell surface during early ferroptosis, where it drives sensitivity by enriching pro-ferroptotic, long-chain polyunsaturated fatty acid phospholipids through a positive-feedback loop. This specificity was confirmed as FABP5 marked damaged neurons in the ischemic penumbra in a mouse model and was significantly elevated in hypoxically damaged human postmortem neurons. These findings establish FABP5 as a robust pathological marker for detecting ferroptosis in human hypoxic/ischemic conditions, offering valuable potential for assessing stroke severity and monitoring therapeutic responses. Furthermore, the discovery of metabolic triggers such as the PPM1K/BCKDHA axis expands the understanding of ferroptosis beyond classic redox pathways, linking branched-chain amino acid metabolism to neuronal vulnerability (Li T., 2023).

Additionally, nuclear factor erythroid 2-related factor 2 (NRF2) activation, as shown in studies involving Icariside II (ICS II) pretreatment, conferred protection by reducing oxidative stress and promoting long-term recovery (Gao, 2023). NRF2 activation orchestrates a coordinated defense against cerebral ischemia by transcriptionally repressing ferroptosis through the GPX4/SLC7A11 axis and iron metabolism regulation, while concurrently mitigating oxidative stress, neuroinflammation, and apoptosis. This integrated mechanism substantiates the therapeutic potential of NRF2 activators like ICS II in fostering neurological recovery (Deng, 2023). Caffeic acid, administered in a permanent MCAO (pMCAO) rat model, was shown to alleviate cerebral ischemic injury in rats by resisting ferroptosis via NRF2 signaling pathway (Li X. N., 2024).

Other components, such as Rehmannioside A, exhibited significant neuroprotective effects by alleviating cognitive impairments and reducing infarct size and neurological deficits (Fu, 2022). Separately, in an MCAO/R rat model, modulation of the HSP90-GCN2-ATF4 pathway was shown to significantly mitigate ferroptosis and necroptosis, suggesting a dose-dependent neuroprotective effect (Zhou Y., 2024). Meanwhile, another study elucidated a distinct mechanism where USP14 deubiquitinates and stabilizes NCOA4, thereby promoting ferritinophagy, increasing free iron, and triggering ferroptosis. Notably, inhibiting either USP14 or NCOA4 blocked this process and protected neurons, identifying this axis as a promising therapeutic target (Li, 2021). Collectively, these discoveries synergize with established targets such as ACSL4 and FABP5, to map a dynamic interplay of redox imbalance, metabolic dysregulation, and cell-type-specific vulnerability, thereby advancing therapeutic strategies for ferroptosis modulation in stroke. A summary of the experimental evidence is provided in Table 1.

**Table 1:** Summary of preclinical evidence.

Species	Experimental models	Mechanism	Reference
Rats	MCAO	G-Rd protects BBB after cerebral ischemia/reperfusion via NRG1/ErbB4-PI3K/Akt/mTOR axis to inhibit endothelial ferroptosis	Hu (2024)
Mice	MCAO	Circ_0008146 exacerbates neuronal ferroptosis in acute ischemic stroke through the miR-342-5p/ACSL4 axis	Liu (2024)
Rats	MCAO, HT-22 cell of OGD/R	NTF protects against CIRI-induced neuronal injury by inhibiting ferroptosis via the HSP90-GCN2-ATF4 pathway	Zhou Y. (2024)
Mice	MCAO	FABP5 levels rise during ferroptosis and target neurons affected by stroke in mice	Peng (2024)
Mice	MCAO, HT-22 cell of OGD/R	Melatonin protects against ischemic stroke-induced ferroptosis through the SIRT6-NCOA4-FTH1pathway	Yu (2024)
Rats	MCAO, SH-SY5Y cell exposed to H_2_O_2_	Rehmannioside A protects against ischemic damage by inhibiting ferroptosis through the PI3K/AKT/NRF2 and SLC7A11/GPX4 pathways	Fu (2022)
Mice	MCAO, Primary astrocyte of OGD/R	Icariside II prevents stroke damage via Nrf2-mediated ferroptosis suppression	Gao (2023)
Rats	MCAO, HT-22 cell of OGD/R	Rhein reduces brain ischemia/reperfusion injury by suppressing ferroptosis via NRF2/SLC7A11/GPX4	Liu (2023)
Mice	MCAO, Primary cortical neuron of OGD/R	NCOA4-ferritinophagy triggers ferroptosis in stroke	Li (2021)

### 3.3 Contradictions and context-dependency in ferroptosis signaling

The emerging evidence linking ferroptosis to ischemic stroke is accompanied by a growing number of seemingly contradictory findings. Systematically deconstructing the origins of these divergent results, including model system disparities, cell-type-specific pathway engagement, temporal dynamics, and methodological variations, is essential to refine our mechanistic understanding and guide successful therapeutic development.

The investigation of ferroptosis in ischemic stroke is fundamentally shaped by the experimental context, where a stark disparity exists between reductionist *in vitro* systems and complex *in vivo* models, a critical consideration for interpreting the literature and guiding translational efforts. *In vitro* models, such as oxygen–glucose deprivation (OGD) in homogenous neuronal cultures, are powerful for deconstructing the core ferroptosis pathway (Ni, 2022). In these systems, core pathway inhibitors like Ferrostatin-1 and Liproxstatin-1 demonstrate robust efficacy by directly scavenging lipid radicals (Chen, 2023a; Zhang, 2023), while agents such as Piceatannol validate the GPX4 axis by enhancing its stability, and Deferoxamine (DFO) effectively chelates intracellular iron (Zhang S., 2022; Li, 2008).

However, the therapeutic efficacy observed *in vitro* frequently encounters significant translational barriers *in vivo*. A potent compound in a dish may fail to cross the blood–brain barrier, be metabolically inactivated systemically, or elicit unexpected off-target effects within the integrated neurovascular unit, where a drug’s action on one cell type can inadvertently impact another, as exemplified by the context-dependent role of molecules like GLRX5 (Zhou S., 2024; Liu, 2024; Liu H., 2025). This principle of context-dependent efficacy is further highlighted by studies on cellular stress response mechanisms (Wang, 2019). For instance, research comparing the differential neuroprotection offered by HSP70-hom gene single nucleotide polymorphisms demonstrated distinct outcomes between *in vitro* neuronal hypoxic injury models and *in vivo* rat middle cerebral artery occlusion models, underscoring how a protective genotype in a controlled cellular environment may not fully recapitulate its effects within the complex pathophysiology of a living organism.

Beyond in vitro or in vivo models, the specific pathophysiological context of the ischemic model itself profoundly influences the observed therapeutic mechanisms and windows. Ischemia–reperfusion (I/R) models, such as transient middle cerebral artery occlusion (tMCAO), are characterized by a robust oxidative burst and a significant exogenous iron load from hemoglobin, creating an environment where interventions like DFO are particularly effective (Hanson, 2009). In contrast, permanent ischemia models lack this iron surge and are defined by sustained metabolic failure, thereby placing greater emphasis on the collapse of endogenous antioxidant systems, making strategies that boost the GSH/GPX4 axis more relevant (Zhang J., 2022). Furthermore, other models like global hypoperfusion highlight white matter injury (Ran, 2020). This model-specificity is a primary reason for the varying reported efficacy of ferroptosis inhibitors across studies.

Compounding this complexity, susceptibility to ferroptosis demonstrates marked heterogeneity across distinct neural cell types. Neurons, with their high metabolic rate and GPX4-dependence, are the primary targets (Yuan, 2021). Astrocytes assume a dual role, supporting neuronal antioxidant defenses while being vulnerable themselves (An, 2025). Microglial ferroptosis amplifies neuroinflammation via ACSL4 and the NLRP3 inflammasome, a mechanism distinct from neuronal death (Zhou, 2023). Endothelial ferroptosis directly disrupts the BBB, initiating a vicious cycle of damage (Zheng, 2022). This cellular heterogeneity explains why studies focusing on different cell types emphasize divergent pathways and underscores the need for cell-type-specific or multi-target therapeutic approaches.

Adding a crucial temporal dimension, the contribution of ferroptosis evolves dynamically post-stroke. The hyperacute phase is characterized by rapid antioxidant failure, creating a narrow window for direct inhibitors (Ginsberg, 2008). However, it remains unclear precisely how the efficacy of a given anti-ferroptotic agent shifts throughout the disease continuum of ischemic stroke. As the injury progresses into the subacute phase, secondary neuroinflammation amplifies and intertwines with ferroptosis, potentially diminishing the efficacy of acute-phase interventions (Bernardo-Castro, 2021). This is exemplified by microglia, which promotes injury not only via acute ferroptosis but also through sustained inflammation, a later-phase response that can render a drug administered only at onset ineffective (Liu Z., 2025). This temporal shift means that studies conducted at different time points capture fundamentally distinct pathological snapshots, favoring treatment strategies that are precisely timed or multi-mechanistic.

Finally, the interpretation of ferroptosis is further complicated by significant methodological heterogeneity. Differences in pharmacological inhibitors, genetic tools (e.g., acute knockdown vs. constitutive knockout), and biomarkers (e.g., non-specific MDA vs. specific lipidomics) mean that studies employing different toolkits are effectively measuring different facets of the process (Chen, 2023a; Cui, 2021; Luo, 2025).

## 4 Therapeutic strategies targeting ferroptosis pathways in ischemic stroke

Excessive ferroptosis exacerbates ischemic neuronal injury through iron overload, lipid peroxidation, and oxidative stress (Guo et al., 2023). To counteract this, therapeutic interventions must balance the dual role of ferroptosis-limiting its pathological effects while preserving its regulatory functions. This section explores pharmacological and innovative strategies to mitigate ferroptosis-driven damage in ischemic stroke, emphasizing emerging therapies and translational opportunities (Summarized in Table 2).

**Table 2:** Therapeutic agents targeting ferroptosis in ischemic stroke.

Therapeutic agent	Mechanism of action	Potential application	Reference
Ferrostatin-1 and Liproxstatin-1	Decreased ferroptosis by scavenging lipid peroxides, blocked lipid peroxidation chain reactions	Therapeutic potential for ischemic stroke	Yang (2022)
rHF nanoparticles	Decreased neuronal ferroptosis, acted as a ROS scavenger	Therapeutic applications in brain ischemia–reperfusion injury	Qi (2024)
TQHX	Enhanced degradation of ACSL4 lowers oxidative stress and prevents ferroptosis	Pathway for therapies in cerebral ischemia–reperfusion injury	Ou (2024)
NecroX-7 and Eriodictyol-7-O-glucoside	Enhanced radical scavenging and prolonged phospholipid bilayer retention	Development of new agents against iron toxicity	Lu (2024)
KBs	Maintained GPX4 levels, suppressed ACSL4 expression, and preserved mitochondrial cristae quantity	Novel therapeutic mechanism in RIPostC treatment for stroke	Huang (2024)
CSO treatment	Mitigated ischemic stroke damage by inhibiting ferroptosis	Neuroprotection in ischemic stroke	Sun (2023)
Rhein	Suppressed ferroptosis via the NRF2/SLC7A11/GPX4 axis	Therapeutic agent in ischemic stroke treatment	Liu (2023)
L-F001	Blocked RSL3-triggered ferroptosis through iron homeostasis preservation and JNK cascade suppression in HT22 cells	Multitarget drug for treating ferroptosis-related diseases	Peng (2022)
Rehmannioside A	Inhibited ferroptosis by activating PI3K/AKT/NRF2 and SLC7A11/GPX4 pathways following cerebral ischemia	Ameliorated cognitive function after ischemic stroke	Fu (2022)
DMF	Suppressed ferroptosis through NRF2/ARE/NF-κB pathway	Ameliorated cognitive deficits in chronic cerebral hypoperfusion rats	Yan (2021)
Piceatannol	Inhibited ferroptosis by targeting the Piceatannol-USP14-GPX4 axis	Therapeutic target in ischemic stroke treatment	Zhao (2025)

### 4.1 Pharmacological inhibitors and modulators

While numerous ferroptosis inhibitors show promise in preclinical studies, their relative efficacy, optimal time windows, and inherent limitations vary considerably, necessitating a critical appraisal. The radical-trapping antioxidants Ferrostatin-1 and Liproxstatin-1 robustly suppress lipid peroxidation and reduce infarct volumes, establishing them as foundational tools in the field (Yang, 2022). However, comparative studies suggest that Liproxstatin-1 may exhibit superior metabolic stability and a longer plasma half-life, potentially translating to a broader therapeutic window than Ferrostatin-1 *in vivo* (Skouta, 2014; Friedmann Angeli, 2014). This distinction is critical, as the narrow temporal window for effective neuroprotection post-stroke demands inhibitors with rapid and sustained action.

Iron chelators such as DFO act proximally by sequestering the catalytic iron required for the Fenton reaction, making them highly effective in the hyperacute phase dominated by iron overload (Moradi, 2025). This is supported by a clinical trial in ischemic stroke patients, which demonstrated DFO’s safety, tolerability, and its mechanism of action in reducing transferrin iron saturation and diminishing the labile iron pool (Millán et al., 2021; Bruzzese, 2023). However, the therapeutic promise of DFO is constrained by a critically narrow window of efficacy and challenges such as poor BBB penetration and potential systemic toxicity. This profile stands in clear contrast to inhibitors like Liproxstatin-1, which targets the lipid peroxidation cascade downstream of iron and may offer a more flexible intervention timeline.

Beyond these classic inhibitors, compounds like NecroX-7 and Eriodictyol-7-O-glucoside, which possess polar structural features, demonstrate efficacy by scavenging ROS and attenuating iron toxicity, yet their blood–brain barrier permeability and precise pharmacokinetics remain less characterized (Lu, 2024). Similarly, indirect modulators like Kinase B Suppressors (KBs), a novel ferroptosis suppressor, target ferroptosis through remote ischemic postconditioning (RIPostC) mechanisms, highlighting its potential for clinical translation (Huang, 2024).

### 4.2 Lipid peroxidation modulation

Targeting lipid peroxidation extends beyond radical scavenging to include enzymatic regulation. A key approach involves inhibiting enzymes like ACSL4, which incorporates polyunsaturated fatty acids into membranes, a critical step for ferroptosis (Gan, 2022). This is exemplified by the small molecule TQHX and rosiglitazone, which promote the degradation or inhibition of ACSL4, thereby reducing lipid peroxidation (Ou, 2024; Li, 2019). This mechanism may confer advantages when upstream radical generation is intractable, though chronic use risks disrupting essential lipid metabolism. Beyond direct enzyme targeting, a complementary strategy involves enhancing the body’s endogenous antioxidant systems. Compounds like Rhein and Reganone achieve this by activating the NRF2 signaling pathway, which upregulates a network of protective genes, including SLC7A11 and GPX4 (Fu, 2022; Liu, 2023). The trade-off lies between the precise action of enzyme inhibitors and the broad, systemic protection offered by NRF2 activators (Xiong, 2023).

### 4.3 Anti-inflammatory and multitarget approaches

Neuroinflammation amplifies ferroptosis via DAMPs and cytokines, creating a vicious cycle of damage (He, 2024). While anti-inflammatory agents like edaravone dexborneol and minocycline can attenuate this inflammatory drive (Zhao, 2007; Zhao, 2024), their standalone efficacy in stroke is often limited. This has spurred the development of intrinsically multi-target agents. L-F001 and dimethyl fumarate (DMF) exemplify this paradigm, simultaneously targeting oxidative stress (including ferroptosis), inflammation (e.g., via NF-κB), and in DMF’s case, even promoting astrocytic health (Peng, 2022; Vanani, 2021) The proven efficacy of DMF in other neuroinflammatory contexts like Alzheimer’s disease reinforces the validity of this pleiotropic strategy (Wang, 2024). The key advantage of these agents is their ability to intervene at multiple nodes of the injury cascade, making them potentially effective across a wider temporal spectrum, especially in the subacute phase where pure ferroptosis inhibitors may fail. Consequently, therapeutic strategies that combine the modulation of pathways like astrocytic glutamate metabolism with antioxidant and anti-inflammatory approaches may offer superior outcomes.

### 4.4 Innovative therapeutic platforms

Emerging therapeutic platforms aim to overcome the limitations of small molecules through enhanced targeting and delivery. Nanoparticle-based systems, such as recombinant human ferritin (rHF), represent a significant advance by functioning as scavengers for multiple types of ROS and inhibiting parallel cell death pathways (Qi, 2024). Their primary advantage lies in their potential for engineered BBB penetration and targeted delivery, which could maximize local efficacy while minimizing systemic exposure. Similarly, gene therapy approaches that upregulate GPX4 or silence ACSL4 offer the ultimate in specificity, allowing for the precise and potentially long-lasting modulation of key ferroptosis nodes (Bai, 2024; Wang, 2022). However, both platforms face substantial translational hurdles, including immunogenicity, manufacturing complexity, and the safe control of transgene expression. While small molecule inhibitors of enzymes like LOXs continue to be explored for their precision (Cakir-Aktas, 2023), the field is increasingly recognizing that the future may lie in these sophisticated platforms capable of delivering therapeutic payloads with unprecedented accuracy to the injured brain.

## 5 Current challenges and future perspectives in ferroptosis research in ischemic stroke

Notwithstanding the compelling evidence linking ferroptosis to ischemic stroke, several formidable challenges impede its therapeutic translation. A primary impediment is the incomplete and often context-dependent understanding of its molecular mechanisms. Beyond the core pathways of iron metabolism and GPX4, the contributions of parallel systems such as FSP1/CoQ10 and GCH1/BH4 in the ischemic brain remain inadequately quantified. Moreover, the field is further complicated by perplexing contradictions, exemplified by the opposing roles of regulators like GLRX5 across different cellular or disease contexts, underscoring that targets cannot be simplistically classified as universally pro- or anti-ferroptotic. Additionally, the precise spatiotemporal crosstalk between ferroptosis, apoptosis, and necroptosis adds a layer of complexity that confounds the design of specific interventions.

To overcome these mechanistic hurdles, future research must prioritize cell-type-specific resolution utilizing advanced tools like conditional knockout models and single-cell omics to map the ferroptosis network within the neurovascular unit. It is equally critical to quantify the relative weight of alternative pathways and decipher the hierarchical interplay of different cell death modalities to identify optimal therapeutic windows and rational combination strategies.

Beyond the mechanistic complexities, the journey from bench to bedside is fraught with additional translational barriers. The profound physiological differences between animal models and humans, combined with the narrow therapeutic window of acute stroke, demand innovative solutions. In particular, the current over-reliance on targeting the GSH/GPX4 axis may be insufficient, especially in prolonged ischemia, while the synergy between ferroptosis, neuroinflammation, and metabolic dysfunction suggests that monotherapies are unlikely to yield robust outcomes. Furthermore, therapies targeting ferroptosis may disrupt iron homeostasis or lipid metabolism, which are crucial for normal physiological function, making the balance between efficacy and safety a key challenge. Iron chelators, for instance, carry the risk of systemic iron depletion and potential anemia, while broad-spectrum lipid peroxidation inhibitors may disrupt essential lipid signaling pathways.

Consequently, to achieve a favorable therapeutic window, future efforts must prioritize strategies that enhance precision and selectivity. This entails: (1) developing brain-targeted delivery systems (e.g., nanoparticles) and context-activated prodrugs to minimize systemic exposure; (2) restricting administration to the acute phase to avoid long-term homeostatic disruption; and (3) pursuing highly selective inhibitors for pathological nodes like ACSL4, or iron-targeting strategies that block the Fenton reaction without depleting essential storage iron, thereby preserving physiological metabolism.

A critical factor complicating this translation is the significant heterogeneity in methodological variables (including the choice of animal species, model type, and ischemia duration) across preclinical studies. For instance, investigations into the efficacy of compounds like Rehmannioside A or the role of HMGB1 in astrocyte-mediated iron dysregulation have been conducted in mice subjected to transient MCAO, albeit with varying occlusion times (e.g., 90 min; Fu, 2022; Davaanyam, 2023). Conversely, studies evaluating the protective effects of agents such as Dihydromyricetin or Butylphthalide frequently utilize rat MCAO models, with ischemia durations ranging from 1 to 3 h (Xie, 2022; Sun, 2024). This heterogeneity in experimental design, spanning different species (mouse vs. rat), ischemia–reperfusion parameters, and the complementary use of *in vitro* models like HT-22 cells or SH-SY5Y cell line under OGD, directly impacts the pathophysiological spectrum of the resulting injury. Consequently, the apparent efficacy of a therapeutic agent and the relative contribution of a specific pathway to ferroptosis can appear context-dependent. Therefore, cross-comparison of findings demands careful consideration of these experimental parameters, and the future development of therapies would benefit from standardized models or studies explicitly designed to evaluate the impact of these variables on ferroptosis.

Finally, to bridge the gap between heterogeneous preclinical models and successful clinical application, a pivotal step toward clinical success lies in advancing precision medicine through biomarker-driven patient stratification. The heterogeneity of the human stroke population necessitates a move beyond a one-size-fits-all approach. Thus, the promising biomarker FABP5, which is specifically elevated in ferroptotic human neurons, must be rapidly validated in clinical cohorts. The immediate goal is to correlate its levels in accessible biofluids with stroke severity and progression, thereby establishing its utility for enriching clinical trials with patients most likely to benefit from ferroptosis inhibition.

This review consolidates the pivotal role of ferroptosis in exacerbating ischemic stroke pathology, driven by iron dysregulation, lipid peroxidation, and mitochondrial collapse. Despite promising preclinical results with inhibitors (e.g., ferrostatin-1) and gene therapies targeting GPX4/ACSL4, clinical translation faces critical barriers: the poorly defined temporal dynamics of ferroptosis post-stroke hinders therapeutic window optimization, and its interplay with necroptosis/pyroptosis requires clarification to guide combination therapies. Future research should prioritize: (1) longitudinal mapping ferroptosis activation in human-relevant models to define temporal therapeutic windows; (2) developing targeted delivery platforms and highly selective inhibitors to enhance efficacy and safety; (3) launching large scale clinical studies that integrate biomarker-driven stroke patient stratification (e.g., using FABP5) with ferroptosis modulation; and (4) rigorous assessment of the long-term systemic risk–benefit profile of these targeted therapies.
